# Design and investigation of the effectiveness of a metatarsophalangeal assistive device on the muscle activities of the lower extremity

**DOI:** 10.1371/journal.pone.0263176

**Published:** 2022-02-10

**Authors:** Jiyoun Kim, Jinkyu Lee, Donghwan Lee, Jiyoung Jeong, Pankwon Kim, Choongsoo S. Shin

**Affiliations:** Department of Mechanical Engineering, Sogang University, Seoul, Republic of Korea; National Tsing Hua University, TAIWAN

## Abstract

The metatarsophalangeal (MTP) joint is not considered in most current walking assistive devices even though it plays an important role during walking. The purpose of this study was to develop a new MTP assistive device and investigate its effectiveness on the muscle activities of the lower extremities during walking while wearing the device. The MTP assistive device is designed to support MTP flexion by transmitting force through a cable that runs parallel with the plantar fascia. Eight participants were instructed to walk at a constant speed on a treadmill while wearing the device. The muscle activities of their lower extremities and MTP joint kinematics were obtained during walking under both actuated and non-actuated conditions. Paired *t*-tests were performed to compare the differences in each dependent variable between the two conditions. The muscle activity of the MTP flexor was significantly reduced during walking under actuated conditions (*p* = 0.013), whereas no differences were found in the muscle activities of other muscles or in the MTP joint angle between actuated and non-actuated conditions (*p* > 0.05 for all comparisons). In conclusion, the cable-driven MTP assistive device is able to properly assist the MTP flexor without interfering with the action of other muscles in the lower extremities; as such, this MTP assistive device, when integrated into existing exoskeleton designs, has the potential to offer improved walking assistance by reducing the amount of muscle activity needed from the MTP flexor.

## Introduction

Various wearable walking assistive devices that improve a person’s ability to walk have been proposed [1–3]. These devices are designed to reduce the effort needed to walk by providing additional torque at the joints of the lower extremities such as at the hip, knee, and ankle joints [4,5], however, the metatarsophalangeal (MTP) joint has rarely been considered in previous assistive devices. The MTP joint has essential function in walking but the importance of this joint during locomotion has been overlooked. The MTP joint supports over 30% of the body mass during the push off phase [6,7] and also contributes to controlling the angular momentum of the whole body in the early stages of the double support phase [8]. As such, absence of MTP joint function or weakness of the MTP joint muscle could cause some biomechanical changes in gait. For example, the loss of MTP joint mobility decreases stride length and walking speed [9]. Inactivity or fatigue of the toe flexor muscles can lead to excessive bending moments on the metatarsals, which, in turn, can lead to metatarsal overuse injuries [10,11]. Thus, one can expect that an effective wearable MTP device, which minimizes the loss of MTP joint function and prevents MTP joint muscle fatigue, can provide effective assistance to locomotion while preventing metatarsal injuries.

Few studies have made efforts to develop an assistive device for the MTP joint [12,13]. Green *et al*. developed an exoskeleton that provides simultaneous passive-elastic assistive joint moments at the ankle and MTP joint [13]. Although they showed that their assistive device reduced the metabolic cost of human walking through a pilot study, they did not differentiate how much MTP assistance contributed to this reduction in metabolic cost. Liu *et al*. designed a foot orthosis with a focus on the MTP joint for patients with MTP joint impairments, however, they did not completely verify the effect of their device on MTP motion or MTP muscles [12]. For these reasons, it is necessary to design a new MTP assistive device and verify whether the device can reduce the burden on the MTP joint.

To design effective assistive devices, it is required to understand which interactions between human and machine are desirable [3]. The device must effectively and safely transmit force to assist the targeted joint. One way to achieve this is to utilize cable-driven mechanisms that have shown they can reduce the overall mass of the device and increase user compliance [14]. In particular, since only the flexion moment is dominant at the MTP joint during walking [15], a cable-driven mechanism seems suited to creating a simple and effective MTP assistive device. Thus, this study aims to develop a new MTP assistive device and evaluate its effectiveness on the muscle activities in the lower extremities during level walking. It was hypothesized that MTP flexor muscle activity would decreases after MTP assistive device is actuated.

## Materials and methods

### Mechanical design

**Fig 1:**
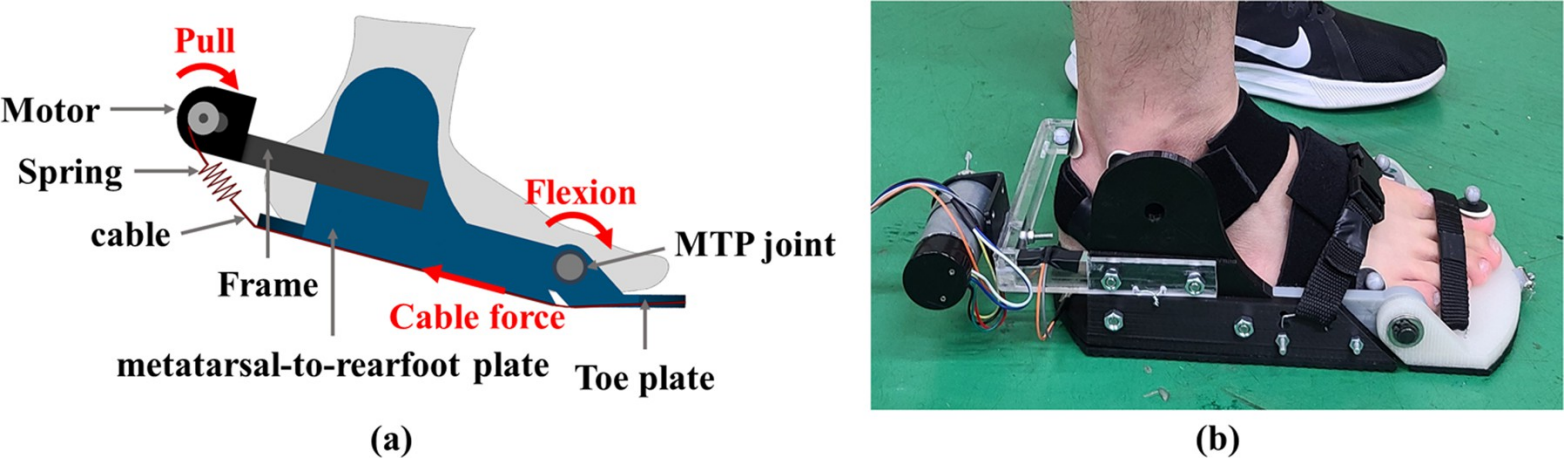
The structure diagram of the MTP assistive device. (A) Schematic diagram; (B) Prototype used in the experiments.

The MTP assistive device assists MTP flexion by transmitting force from a motor through a steel cable to the user’s MTP joint (Fig 1(A)). The device consists of a toe plate, a metatarsal-to-rearfoot plate, and a frame on which the motor is mounted. These two plates are connected via hinges and the center of the joint is 20 mm from the bottom of these plates. One end of the cable is fixed to the front of the toe plate, the other end of the cable passes through a longitudinal slot under the two plates and is connected to a spring located directly behind the user’s heel. Finally, the spring, which provides compliance by acting as a tendon, is connected to the pulley of the motor. The plates have rubber soles with a ripple pattern to provide shock-absorption and grip on slippery surfaces. Users could fix their feet to the device using neoprene straps at three locations: toes, metatarsals, and heel.

Since the MTP flexion moment has a single peak during late stance phase of walking [15], the assistive torque of the device was designed to mimic this using a single peak profile. Due to the lack of research on MTP joint assistive devices, the required peak assistive torque was determined based on a previous study for an ankle exoskeleton device [16]. Collins and colleagues reported that the net metabolic rate was significantly reduced when the peak device torque was about 16% of the peak biological ankle moment. Bruening and Takahashi reported that the peak MTP moment for a healthy adult during walking was about 0.06 Nm/kg [15]. Thus, the required peak assistive torque for our device was determined to be 0.0096 Nm/kg (16% of the peak MTP moment), which was normalized to body weight. Based on this the device is setup to provide a peak assistive torque of 0.864 Nm allowing it to support users up to a maximum weight of 90 kg. Since the length of the moment arm *r* at the MTP joint of the device is 20mm, the required peak cable force *F_assist_* is calculated as *F_assist_* = *τ_assist_*/*r* where *τ_assist_* is the required peak assistive torque. A geared DC motor (24 V, 530 rpm, JGB37-3530) and a spring with stiffness of 1.53 N/mm were used to generate the required peak cable force. A 3D-printed prototype of the MTP assistive devices was manufactured using PLA and resin (Fig 1(B)). Three different sizes of the device for foot lengths of 255 mm, 265 mm, and 275 mm were prepared. The device allows a range of motion (ROM) from 0° to 60° of MTP extension and the total mass of the device, excluding the controller and the battery, for a foot length of 275 mm is 0.985 kg.

### Control

**Fig 2:**
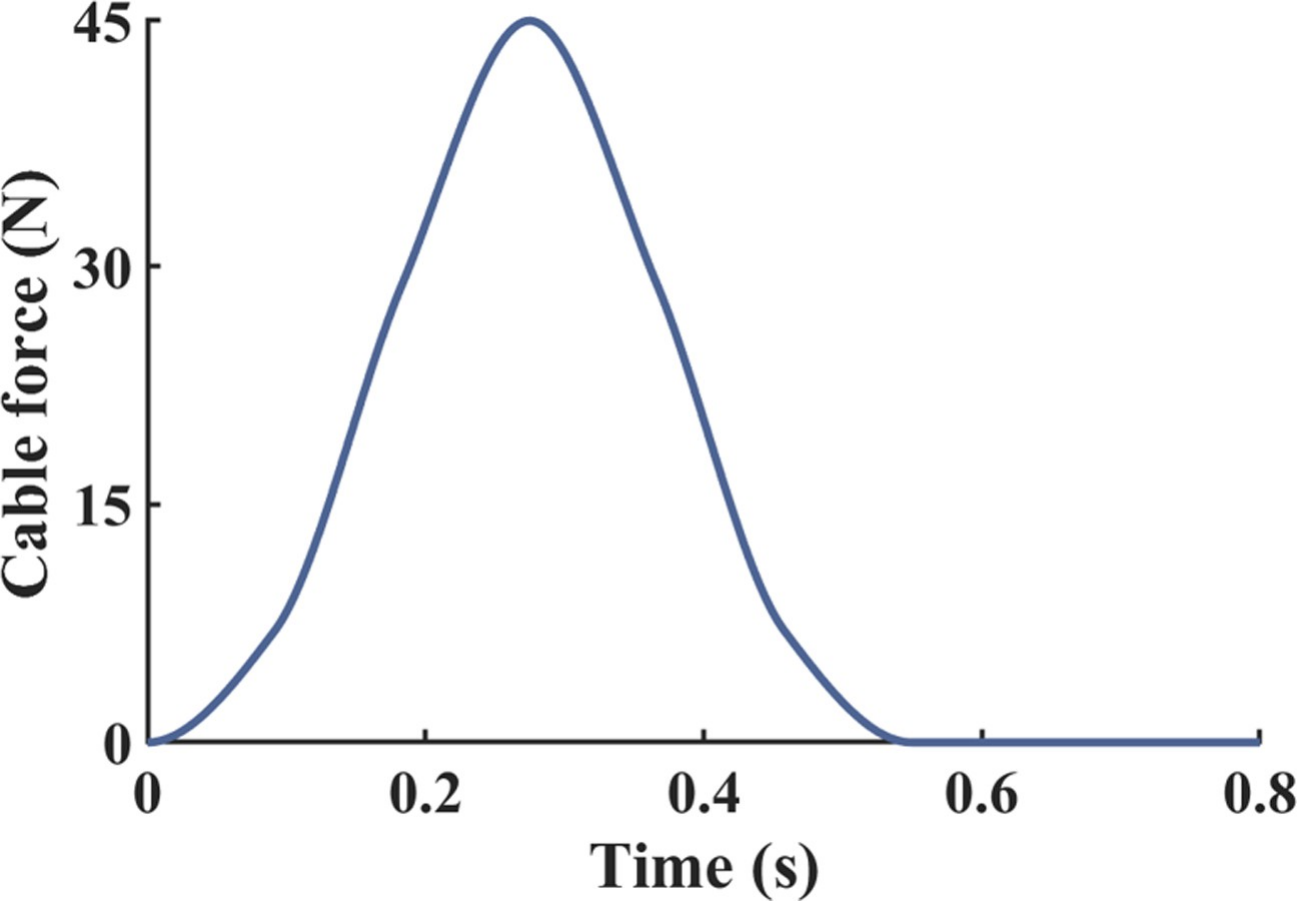
Profile of the cable force. A profile of the assistive cable force was used to determine the actuation onset timing.

**Fig 3:**
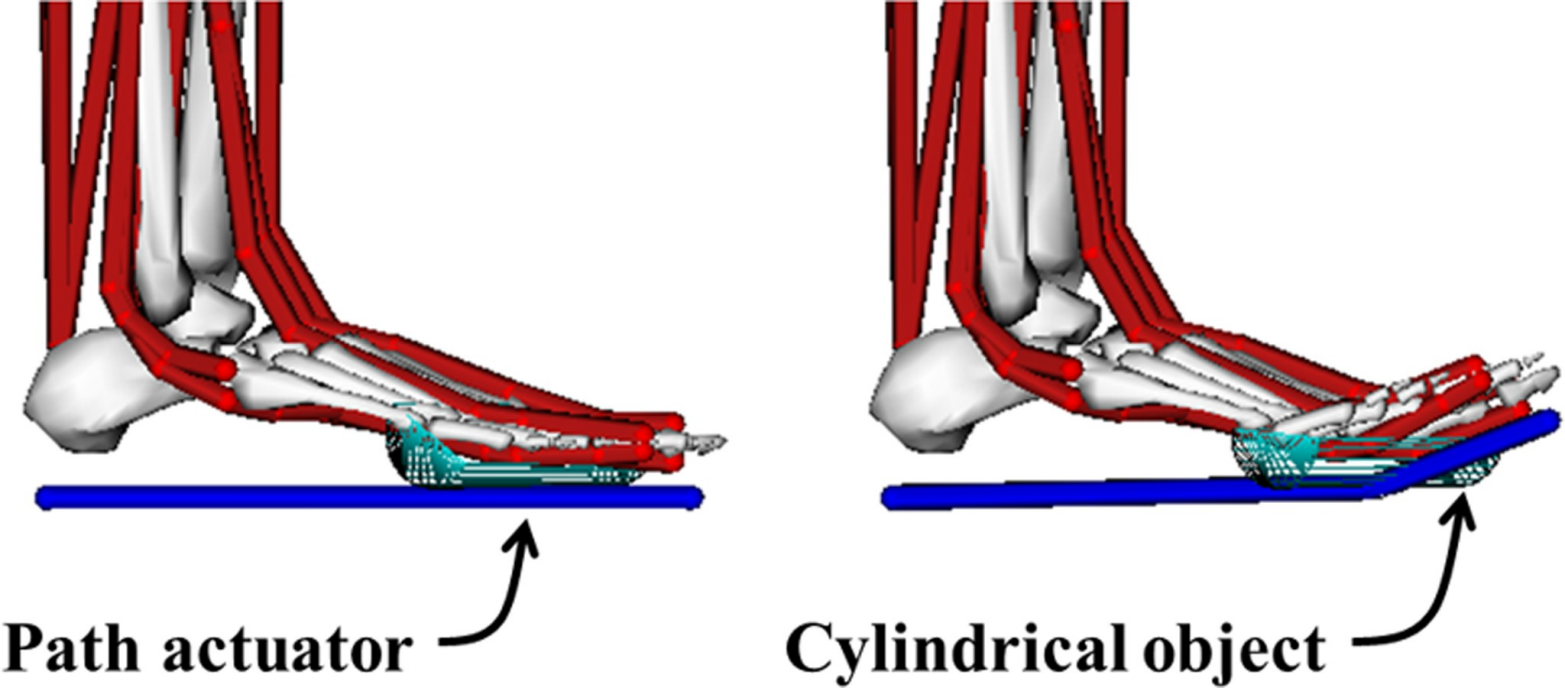
Foot segments of a musculoskeletal model. A path actuator and cylindrical object added to the foot segments of a musculoskeletal model.

**Fig 4:**
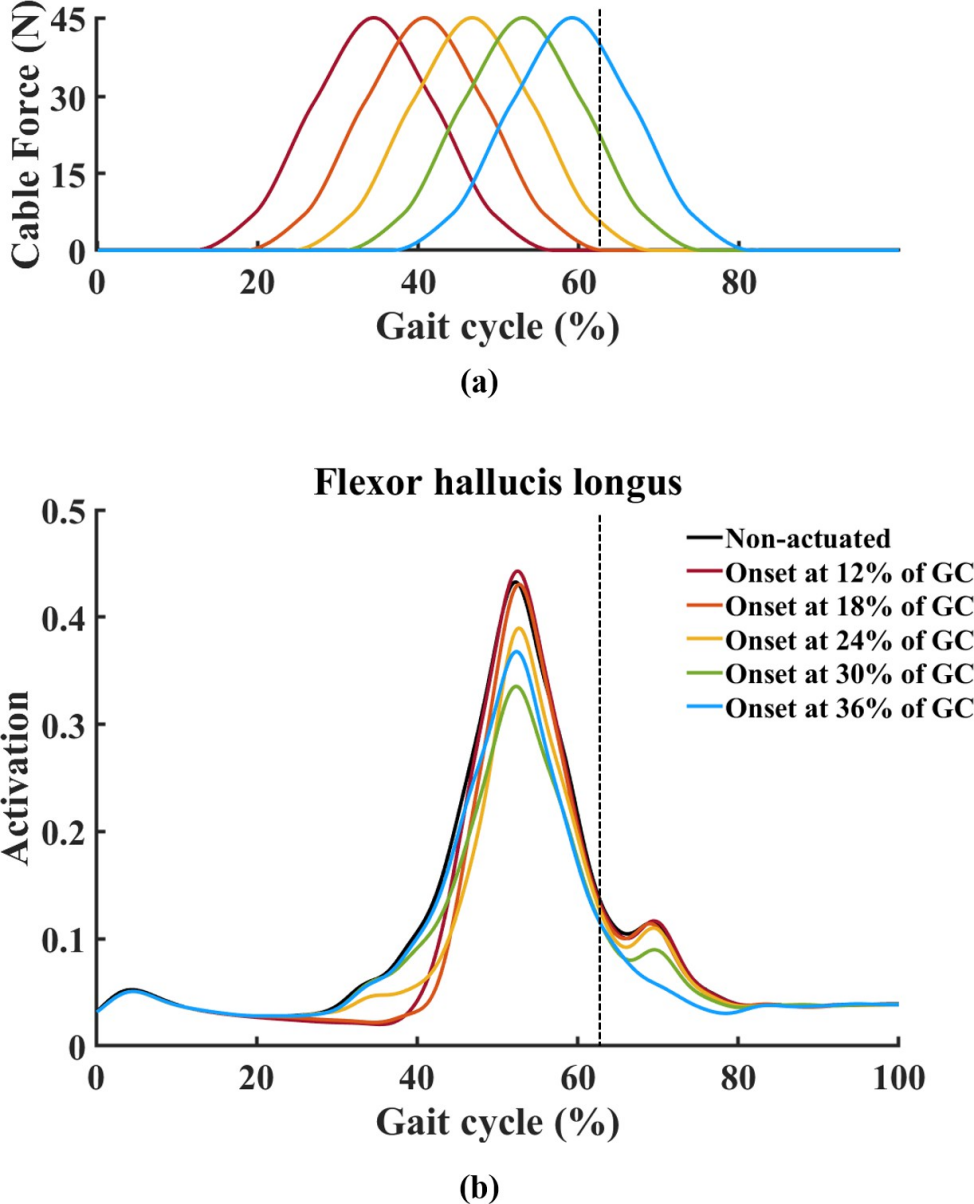
Five onset times and results for musculoskeletal simulation. (A) The five different onset times were at 12%, 18%, 24%, 30%, and 36% of the gait cycle. (B) The results of the simulations showed that the flexor hallucis longus activation was decreased the most compared to the flexor hallucis longus activation without the actuator when the onset timing was set to 30% of gait cycle. The dashed vertical line indicates toe-off event timing.

A motor driver (MD10C, Cytron Technologies, Kuala Lumpur, Malaysia) was utilized to control the direction and velocity of the motor. Using a mechanical test setup with a load cell, a profile of the assistive force provided by the cable according to the motor input was obtained (Fig 2). Since a spring was included in the mechanical test setup, physical characteristics of the spring were reflected in the profile of the assistive force. This profile was used to help determine the actuation onset timing, that is, the time at which the motor starts pulling the cable. In addition to considering the cable force profile to determine the onset timing, further information from a musculoskeletal model simulation in the open-source software OpenSim was also considered [17]. A path actuator was added under the foot in our basic OpenSim model (Fig 3) [18], this actuator receives a signal to control its movement that changes over time according to which part of the gait the user is in. A cylindrical object with a diameter of 20mm was inserted into the MTP joint so the path actuator smoothly wraps over the joint. Motion capture and ground reaction force data were collected from a single male subject (age: 25 years, weight: 80 kg, height: 1.79 m, BMI: 25.0 kg/m^2^) while he was walking at a self-selected speed, this data was used as the input to generate muscle-driven simulations in the Computed Muscle Control tool. Firstly, a simulation without an actuator was conducted, then simulations with an actuator using five different onset times were carried out for each condition. The onset times used were at 12%, 18%, 24%, 30%, and 36% of the gait cycle (GC) (Fig 4(A)). The results of the simulations showed that the flexor hallucis longus (FHL) activation was decreased the most compared to the FHL activation without the actuator when the onset timing was set to 30% of GC (Fig 4(B)). Therefore, the actuation onset timing was determined to be at 30% of the GC. The assistive torque was controlled using PWM (pulse-width modulation), which defined the motor speed. The peak assistive torque for individuals was applied by adjusting the PWM value (76% to 99% of duty cycle) on the control unit based on their body weights.

A pressure sensor (FSR 402, Interlink Electronics, Camarillo, CA, USA) was attached under the heel to detect heel strikes. When a heel strike was detected, the actuator started to pull the cable at 30% of the GC to provide assistive torque to the MTP joint. After the force provided by the assistive cable reached its maximum value, the cable was released by rotating the motor in the opposite direction at the same speed as it was pulled and returned to its initial position. The device is then ready to detect the next heel strike as the next GC begins.

### Experiments

Eight male subjects with no previous lower extremity injuries (age: 28.8±5.7 years, mass: 73.9±11.8 kg, height: 1.72±0.06 m, BMI: 24.9±4.2 kg/m^2^) participated in the experiments after signing an informed consent document approved by the Institutional Review Board. Participants wore the MTP assistive device on their bare right foot and walked at 3.6 km/h on a treadmill for all tests (Fig 1(C)). Prior to the actual tests, participants were given an approximately 15 min to walk to get used to the device. The average GC of each subject was also measured to calculate the 30% of the GC. This calculated time was used as the constant onset timing in the test session individually. The height of the right foot wearing the device was matched to the height of the left foot wearing a sneaker. During the test session, the participants first warmed up while not wearing the device [19]. After resting for about 5 minutes, participants walked for 2 minutes while wearing the device under non-actuated conditions (i.e. the device was powered off). The cable was loosened so that the tensile force of the spring would not occur when participants walked. After another 5-min break, participants walked for 2 minutes while wearing the device under actuated conditions (i.e., with the motor providing assistance to the MTP joint). At this time, the cable was set to taut without the spring stretched. As soon as the speed of the treadmill reached the target speed, the device was powered on.

**Fig 5:**
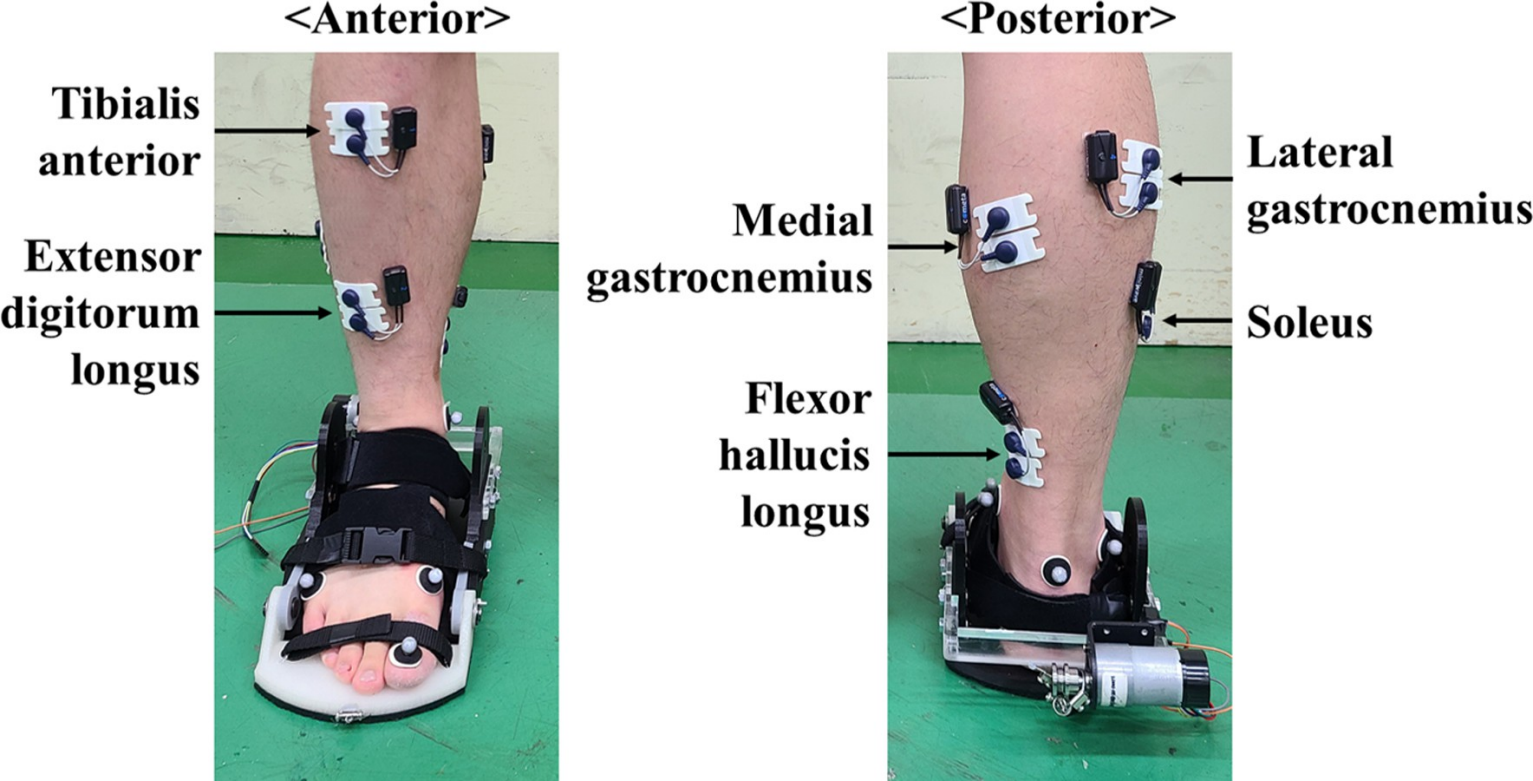
Placement of the electrodes on the right ankle and MTP muscles. Surface EMG signals from the flexor hallucis longus, extensor digitorum longus, tibialis anterior, gastrocnemius medialis, gastrocnemius lateralis, and soleus were collected during the tests.

A motion capture system with 10 infrared cameras (9 Eagle, 1 Raptor; Motion Analysis Corp., Santa Rosa, CA, USA) was used to record the positions of six reflective markers placed on the heel, lateral malleolus, medial malleolus, first metatarsal head, fifth metatarsal head, and the big toe of the participants at a sampling rate of 400 Hz. A wireless EMG system (Cometa, Milan, Italy) was used to record surface EMG signals from the FHL, extensor digitorum longus (EDL), tibialis anterior (TA), gastrocnemius medialis (GM), gastrocnemius lateralis (GL), and soleus (Sol) with a sampling rate of 1200 Hz (Fig 5). The electrodes were placed in accordance with recommendations from previous studies [20–22].

### Data processing

The motion capture data were filtered using a zero-lag fourth-order Butterworth filter with a cut-off frequency of 10 Hz. To calculate the MTP joint angle, the coordinate system for the toe and metatarsal-to-rearfoot segment were defined following the methods outlined in previous studies [23,24].

The raw EMG data were filtered with a band pass Butterworth filter at 20–460 Hz, fully rectified and low-pass filtered with a cut-off frequency of 6 Hz [16]. The processed EMG data were then normalized to the average peak value per stride observed during the tests under non-actuated conditions for each muscle of each participant [25]. The mean EMG values for each muscle were calculated over the last 10 strides for each participant [26]. Heel strikes were determined by identifying the moment the heel marker begins moving backward [27]. The toe-off was identified by when the vertical velocity of the toe marker went over 100 mm/s [28]. The stride length was calculated as the product of mean stride time and walking velocity [29].

### Statistical analysis

Paired *t*-tests were performed to verify the effects of the MTP assistive device on MTP flexor muscle activity, by comparing the mean and peak muscle activities between two conditions: actuated and non-actuated. The differences in stance time, swing time, stride length, MTP ROM, and MTP extension/flexion angle between the two conditions were also checked by performing another set of paired *t*-tests. The significance level for all tests was set to α = 0.05. All statistical analyses were conducted using MATLAB version R2017b (MathWorks, Natick, MA, USA).

## Results

**Fig 6:**
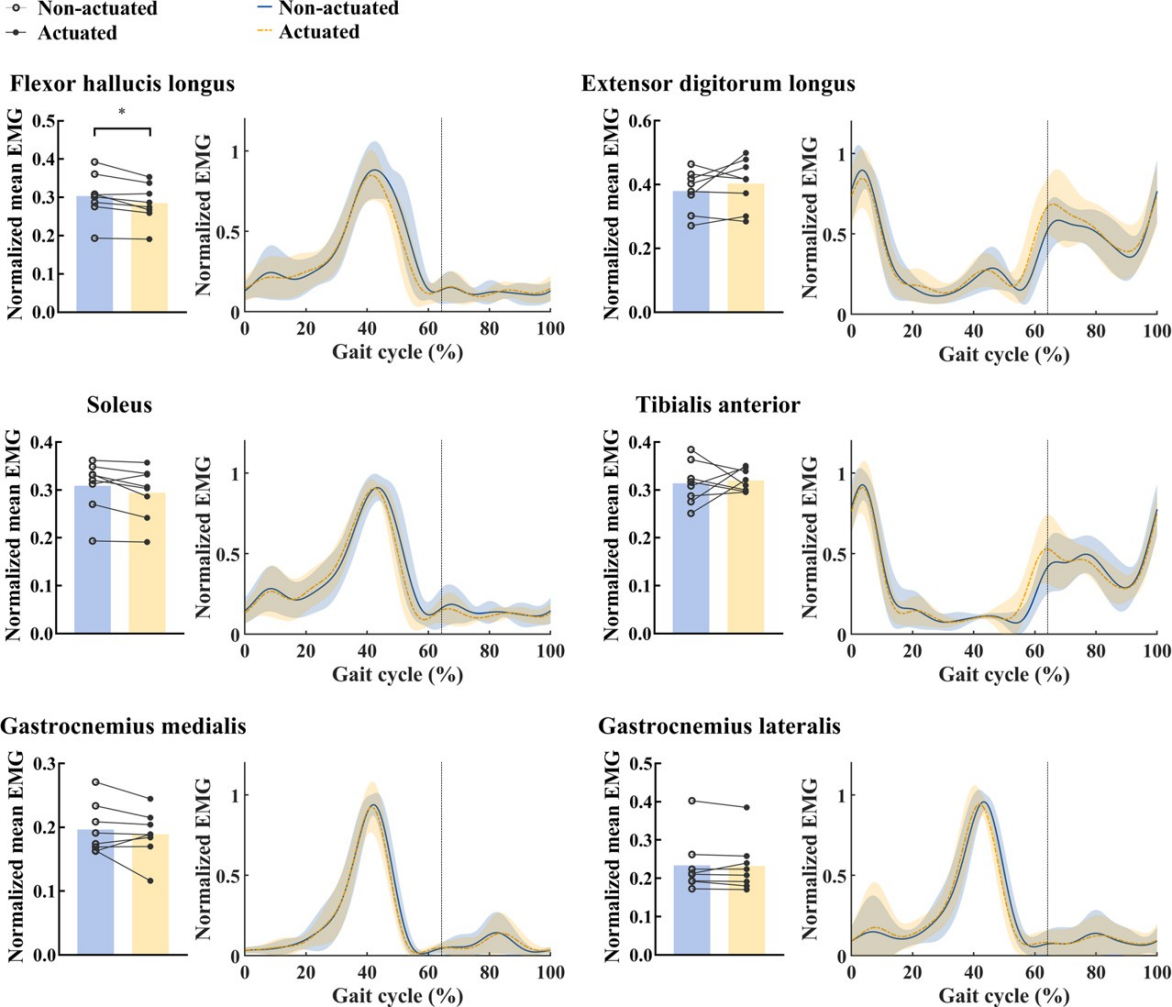
Muscle activities of six muscles over the one gait cycle. Normalized mean EMG (left) and normalized EMG over one gait cycle (right) under actuated and non-actuated conditions are shown for each muscle. Lines link data points from same participant. Curves are averages taken over all participants; shaded regions indicate one standard deviation. The vertical lines indicate the toe-off.

The normalized mean activity of the FHL during walking under actuated conditions show a significant reduction of 6.1% relative to under non-actuated conditions (*p* = 0.013, Fig 6 and Table 1), in contrast, no differences were found in other muscles between actuated and non-actuated conditions (TA: *p* = 0.752; EDL: *p* = 0.284; GM: *p* = 0.367; GL: *p* = 0.710; Sol: *p* = 0.071, Fig 6 and Table 1). The muscle activities of six muscles over the one gait cycle between actuated and non-actuated conditions were compared and illustrated in Fig 6. All EMG values of these muscle activities are summarized in Table 1.

**Table 1:** Means, standard deviation (SD) and statistical results for normalized muscle activities during walking with the MTP assistive device under actuated and non-actuated conditions.

Muscle	Non-actuated		Actuated		*P value*
	mean	SD	mean	SD	
Flexor hallucis longus	0.303	0.059	0.285	0.051	0.013*
Extensor digitorum longus	0.380	0.065	0.403	0.079	0.284
Tibialis anterior	0.314	0.044	0.320	0.023	0.752
Soleus	0.309	0.054	0.294	0.054	0.071
Gastrocnemius medialis	0.197	0.039	0.189	0.037	0.367
Gastrocnemius lateralis	0.234	0.073	0.232	0.069	0.71

* P ≤ 0.05.

**Fig 7:**
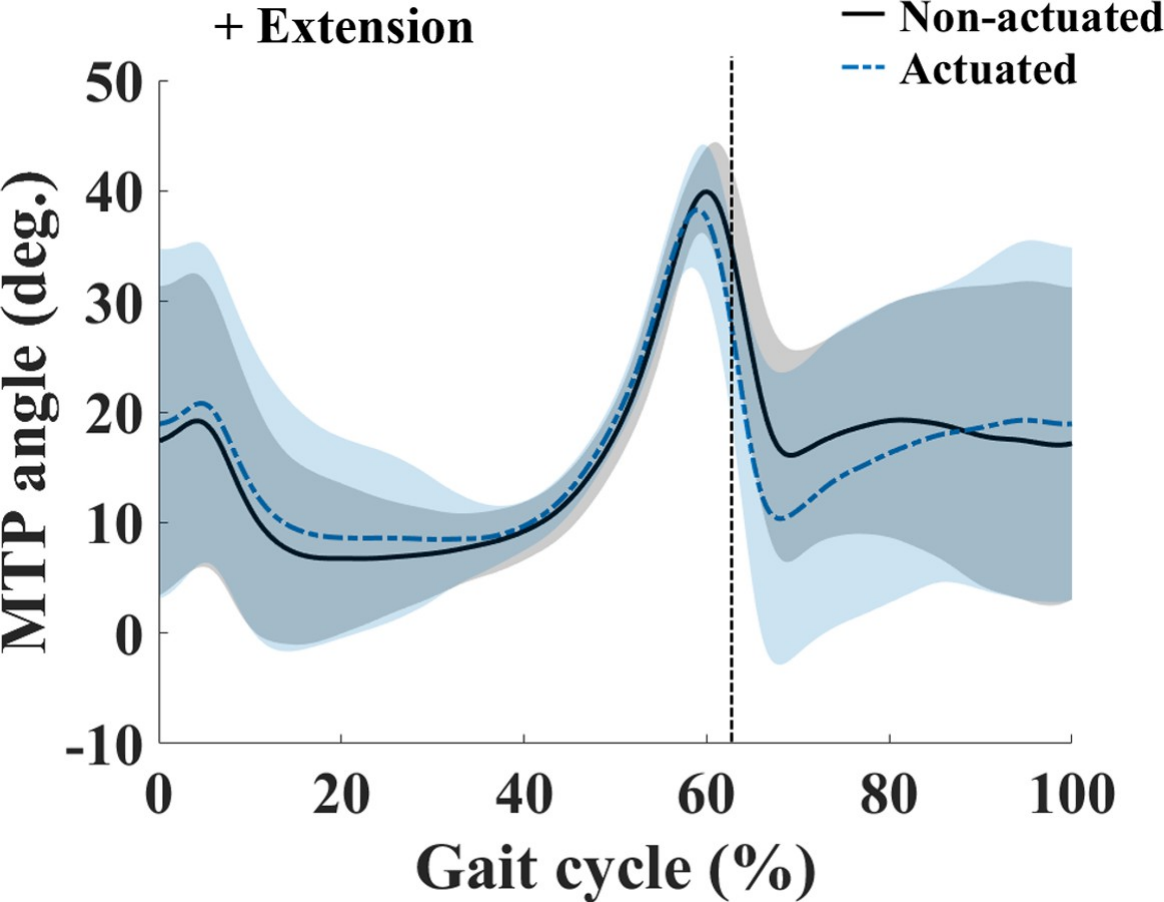
MTP angle over one gait cycle. Curves are averages taken over all participants; shaded regions indicate one standard deviation. The vertical lines indicate the toe-off.

The MTP kinematics over the one gait cycle with the MTP assistive device between actuated and non-actuated conditions were compared and illustrated in Fig 7. In general, the MTP angle shows the single distinctive peak before toe-off during walking. In terms of the observed kinematics, under non-actuated condition, the MTP ROM was 37.5 ± 4.9° and the maximum MTP extension angle was 40.3 ± 3.6° at 60% of GC (Fig 7 and Table 2). Under actuated condition, the MTP ROM was 37.0 ± 6.7° and the maximum MTP extension angle was 39.0 ± 5.3° at 59% of GC (Fig 7 and Table 2). There were no significant differences in MTP ROM, maximum MTP extension angle, and average MTP extension angle during the stance/swing phases between the two conditions (Table 2). The average stride length during walking under non-actuated condition across all participants was 1.194 ± 0.059 m, with a stance time of 0.855 ± 0.033 s and a swing time of 0.339 ± 0.031 s (Table 2). The average stride during walking under actuated condition across all participants was 1.198 ± 0.055 m, with a stance time of 0.858 ± 0.028 s and a swing time of 0.340 ± 0.029 s (Table 2). No significant differences were found in stance time, swing time, and stride length between two conditions (Table 2).

**Table 2:** Means, standard deviation (SD) and statistical results for spatiotemporal and kinematics parameters during walking with the MTP assistive device under actuated and non-actuated conditions.

Parameter	Non-actuated		Actuated		*P value*
	mean	SD	mean	SD	
stance time (s)	0.855	0.033	0.858	0.028	0.516
swing time (s)	0.339	0.031	0.340	0.029	0.839
stride length (m)	1.194	0.059	1.198	0.055	0.677
MTP ROM (°)	37.5	4.9	37.0	6.7	0.715
Max. MTP extension angle (°)	40.3	3.6	39.0	5.3	0.174
Avg. MTP extension angle during stance phase (°)	13.9	4.6	15.1	5.6	0.083
Avg. MTP extension angle during swing phase (°)	18.3	10.2	17.3	11.9	0.6527

## Discussion

The purpose of this study was to propose the cable-driven powered MTP assistive device and examine its effectiveness on the lower leg muscle activity during walking. In order to properly assist MTP flexion, a steel cable was used to transmit assistive torque generated by an actuator to the toe plate of the MTP assistive device, the cable rotates the toe plate in the direction of flexion reducing the burden on the user. This cable-driven mechanism is relatively simple, and the weight of the cable is insignificant relative to the weight of the device, however, this approach is only able to provide a pulling force. Practically, the MTP extension moment can barely be observed during walking, so a force to rotate the toe plate back in the opposite direction seems unnecessary in the MTP assistive device. This MTP assistive device equipped with the cable-driven mechanism has the potential to be applied to existing exoskeletons in a modular manner.

The normalized mean FHL activity during walking decreased significantly under actuated conditions compared to non-actuated conditions. This result supports the hypothesis that the MTP assistive device would decrease MTP flexor muscle activity. The FHL originates from the posterior surface of the fibula and inserts onto the plantar surface of the big toe [30], it produces a flexion moment at the MTP joint [31]. The measured reduction in FHL activity indicates that our device can partially replace the FHL muscle contractions to give effective assistance to the user by reducing the effort required for them to walk. This explanation indicates that this device could also help the toes control the body’s forward motion during the terminal stance phase [32]. To our knowledge, this is the first study to demonstrate a reduction in FHL muscle activity during walking with a wearable MTP assistive device.

The results of this study showed there were no significant differences in EDL and ankle muscle activity between actuated and non-actuated conditions. This suggests that our device assists the toe flexor (i.e. FHL) without interfering with the action of the ankle flexor (i.e. TA), ankle extensors (i.e. GM, GL, Sol) or toe extensor (i.e. EDL) during walking. When excessive assistive torque is provided, unnecessary coactivation of the antagonist muscles could be induced to counteract this torque [19]. As such, the results from this study imply that our device is properly designed and effective in assisting MTP flexion during walking.

It is notable that there was no change in the average MTP extension angle during the swing phase between actuated and non-actuated conditions. During the swing phase, MTP extension of more than 10° occurs to provide toe clearance [33], adequate toe clearance is essential to avoid tripping and falling [34]. To achieve this toe clearance, proper MTP extension with ankle dorsiflexion and knee flexion is required [35]. In our MTP assistive device, the cable is released enough and provides the range of motion to permit MTP extension before the swing phase begins so the required toe clearance can be achieved.

The MTP ROM, stance time, swing time, and stride length during walking under actuated conditions were not altered when compared to non-actuated conditions. This finding agrees on the results of a previous study, which reported that the restriction of MTP motion affects the spatiotemporal parameters [9]. As the MTP assistive device does not interfere with MTP motion during the swing phase, the gait parameters may not be altered when the MTP assistive device is used. Thus, the results of our study confirm that our device does not interfere with MTP motion and is able to preserve natural walking when wearing the MTP assistive device.

This study has some limitations. First, the assistive torque profile used in our device was symmetric about the peak (Fig 2). In fact, the MTP moment has been observed to have an asymmetric profile that increases gradually early in the stance phase and then rapidly decreases after the peak [15]. It would be advantageous for the assistive torque from our device to follow the pattern of the MTP moment as closely as possible. However, the MTP moment during the early half of the stance phase is so small that even a small increase in assistive torque may negatively affect muscle activity. Consequently, a symmetric assistive torque during second half of the stance phase was chosen and proved sufficient to assist the MTP flexion moment during walking. Second, the MTP joint axis of the device does not exactly align with the functional MTP joint axis. The functional MTP joint axis is rotated by -8° on a right-handed vertical axis at the second metatarsal head [36,37]. The MTP joint axis of the assistive device is aligned in parallel to the mediolateral axis because the joint of the device was designed as a hinge joint. For that reason, the 4th to 5th toes do not function properly while walking in the device. However, when compare the plantar pressure measured during the push-off phase, the force under the big toe and 2nd toe account for 86% of the overall force [6]. Therefore, the restricted function of the 4th to 5th toes may not affect the results of the current study. In future, it is warranted to recruit older participants or MTP impaired subjects to test in order to examine the usefulness of our device among its target users. In particular, user satisfaction assessment and user comfort assessment for people with reduced mobility will be needed to improve the experimental protocol and the quality of the device. In addition, it would be worthwhile to verify the effectiveness of our device while walking in other environments, including uphill and downhill walking, as well as transitions between walking and other activities (e.g., sitting and standing).

## Conclusions

This study demonstrated that the cable-driven MTP assistive device assists the MTP flexor effectively without interfering with MTP motion or the action of other muscles in the lower extremities during walking. The muscle activity of FHL, which is one of the major MTP flexors, significantly decreased when using the device, at the same time the activities of other muscles in the lower extremities were not affected. These results support for the effectiveness of our device to assist the MTP joint during walking and its potential use to improve existing multi-joint exoskeletons in a modular manner could further ease the burden of walking.

## Supporting information

S1 File Dataset. (XLSX)
